# Modified Hemocyanins from *Rapana thomasiana* and *Helix aspersa* Exhibit Strong Antitumor Activity in the B16F10 Mouse Melanoma Model

**DOI:** 10.3390/md22100462

**Published:** 2024-10-07

**Authors:** Emiliya Stoyanova, Nikolina Mihaylova, Nikola Ralchev, Silviya Bradyanova, Iliyan Manoylov, Yuliana Raynova, Krassimira Idakieva, Andrey Tchorbanov

**Affiliations:** 1 Department of Immunology, Stefan Angeloff Institute of Microbiology, Bulgarian Academy of Sciences, 1113 Sofia, Bulgaria; stoyanova_e@microbio.bas.bg (E.S.); mihaylova_n@microbio.bas.bg (N.M.); nikola_ralchev@microbio.bas.bg (N.R.); silvybradyanova@microbio.bas.bg (S.B.); iliyanmanoylov@microbio.bas.bg (I.M.); 2 Institute of Organic Chemistry with Centre of Phytochemistry, Bulgarian Academy of Sciences, 1113 Sofia, Bulgaria; yuliana.raynova@orgchm.bas.bg (Y.R.); idakieva@orgchm.bas.bg (K.I.)

**Keywords:** C57BL/6 mouse melanoma model, modified hemocyanins, anticancer therapy

## Abstract

Melanoma is one of the most common tumors worldwide, and new approaches and antitumor drugs for therapy are being investigated. Among the promising biomolecules of natural origin for antitumor research are gastropodan hemocyanins—highly immunogenic multimeric glycoproteins used as antitumor agents and components of therapeutic vaccines in human and mouse cancer models. A murine melanoma model established in C57BL/6 mice of the B16F10 cell line was used to study anticancer modified oxidized hemocyanins (Ox-Hcs) that were administered to experimental animals (100 μg/mouse) under different regimens: mild, intensive, and with sensitization. The solid tumor growth, antitumor response, cell infiltration in tumors, and survival were assessed using flow cytometry, ELISA, and cytotoxicity assays. Therapy with Ox-RtH or Ox-HaH resulted in the generation of enhanced specific immune response (increased levels of tumor-infiltrated mature NK cells (CD27+CD11b+) in sensitized groups and of macrophages in the intensively immunized animals) and tumor suppression. Beneficial effects such as delayed tumor incidence and growth as well as prolonged survival of tumor-bearing animals have been observed. High levels of melanoma-specific CTLs that mediate cytotoxic effects on tumor cells; tumor-infiltrating IgM antibodies expected to enhance antibody-dependent cellular cytotoxicity; type M1 macrophages, which stimulate the Th1 response and cytotoxic cells; and proinflammatory cytokines, were also observed after Ox-Hcs administration. The modified Hcs showed strong antitumor properties in different administration regimens in a murine model of melanoma with potential for future application in humans.

## 1. Introduction

Skin cancer is the most common type of cancer, with diverse forms of manifestation. The uncontrolled and invasive proliferation of melanocytes can lead to the development of melanoma, an exceptionally aggressive form of skin cancer with heightened metastatic potential. Extensive examination of animal models of melanoma has disclosed that this specific type of cancer is incited by a confluence of genetic background, diverse oncogenes, and signaling pathways, which are modulated by the microenvironment and exacerbated by ultraviolet radiation (UVR) [1,2,3]. Several therapeutic approaches targeting melanoma have been established over recent decades, encompassing surgical resection, chemotherapy, radiotherapy, targeted therapies (involving BRAF, MEK, HDAC, or EZH2 inhibitors), immunotherapies (such as IFN-α, IL-2, IL-10, IL-15, and immune checkpoint inhibitors), nano-therapies, and oncolytic virotherapy [4]. While conventional treatment modalities, such as surgery and radiotherapy, have proven to be efficacious, their application is confined to early stage localized melanoma tumors. Conversely, alternative strategies have exhibited relatively limited efficacy, primarily arising from the inherent chemoresistance of melanoma cells. Therefore, there is an urgent need for more effective and less toxic anticancer drugs for melanoma [5].

Natural products, particularly those derived from marine sources, contain a vast array of chemical compounds that can significantly impact the development of anticancer drugs. The unique structures of these compounds are often associated with specific mechanisms of action that trigger unexpected effects. Accordingly, they can directly affect tumor processes at the cellular and tissue level by blocking various growth factors and mechanisms. Modern medicine desires molecules that enhance anti-tumor action and exhibit limited adverse reactions [6].

Hemocyanins (Hcs) are large oligomeric oxygen-transporting proteins in the hemolymph of mollusks and arthropods. Their molecular size ranges from 3.3 to 13.5 MDa, which is the largest known protein size in nature. Beyond the biological function of Hcs related to oxygen binding, this protein family has shown great potential for use in various clinical trials, including those examining cancer immunotherapy, antiviral agents, protein carriers, and vaccine adjuvants. Hcs are complex glycoproteins that have been found to exhibit a host of beneficial proinflammatory effects as adjuvants in the early stages of immune responses. Notably, they have been shown to induce a strong humoral and cellular immune response, as well as display significant antitumor properties in mammals [7].

The best-studied Hc is the protein keyhole limpet hemocyanin (KLH) isolated from the marine gastropod Megathura crenulata. KLH has been used for decades as an adjuvant, a protein carrier and an anti-cancer drug in various applications [7]. Another Hc isolated from Concholepas concholepas (CCH) was found to have antitumor properties comparable to KLH, while Hcs isolated from Fissurella latimarginata (FLH) and Haliotis tuberculate (HtH) showed better anti-tumor properties to KLH in cancer models [8,9,10,11].

Hcs from the marine snail *Rapana thomasiana* (RtH) and the terrestrial snail Helix pomatia (HpH) have been shown to elicit strong antiviral or antibacterial immune responses in mouse models when combined with bacterial and viral antigens. Moreover, these Hcs have demonstrated potent in vivo anti-cancer and anti-proliferative effects in the murine model of colon carcinoma [12,13,14,15].

Several Hcs, such as CCH, FLH, RtH, and the one isolated from the hemolymph of *Helix aspersa* (HaH), have been found to display remarkable antitumor effects as carriers of tumor-associated mimotopes of gangliosides GD2 and GD3 in a murine model of melanoma [16,17]. Immunization of mice with RtH or HaH alone generated high titers of melanoma-specific IgM antibodies and tumor-specific CTLs in the same B16F10 tumor model [18].

Ensuring the structural stability of proteins is a pivotal stage in the development of effective treatments. Despite the existing knowledge of the glycosylation profile of KLH, its role in immunogenicity and antigenicity is yet to be fully clarified. Previous studies have suggested the presence of carbohydrate epitopes in KLH that cross-react with tumor antigens, resulting in an observed antitumor effect. Research indicates that the oxidation of carbohydrate chains in hemocyanins with sodium periodate leads to enhanced structural stability, thereby increasing resistance to proteolytic degradation and improving thermal stability. By employing oxidation with sodium periodate to modify hemocyanins, a more comprehensive understanding of the involvement of carbohydrate structures in their immunostimulatory and antitumor properties can be attained [8,19,20,21,22]. Our current research objectives are to investigate the immunological properties of modified Hcs (Ox-RtH and Ox-HaH) in the B16F10 murine melanoma model.

## 2. Results

### 2.1. Preparation of Ox-RtH and Ox-HaH

The isolation and purification of Ox-RtH and Ox-HaH were conducted using pyrogen-free materials and reagents. The final solutions contained 14.4 mg/mL Ox-RtH and 8.3 mg/mL Ox-HaH. Subsequent analysis of the final sterile Hc preparations by limulus amebocyte lysate (LAL) assay indicated the presence of endotoxins at levels of 0.9 EU/mg protein for Ox-RtH and 0.7 EU/mg protein for Ox-HaH.

### 2.2. Tumor Development and Survival Analysis after Ox-Hcs Therapy in the B16F10 Murine Melanoma Model

**Figure 1:**
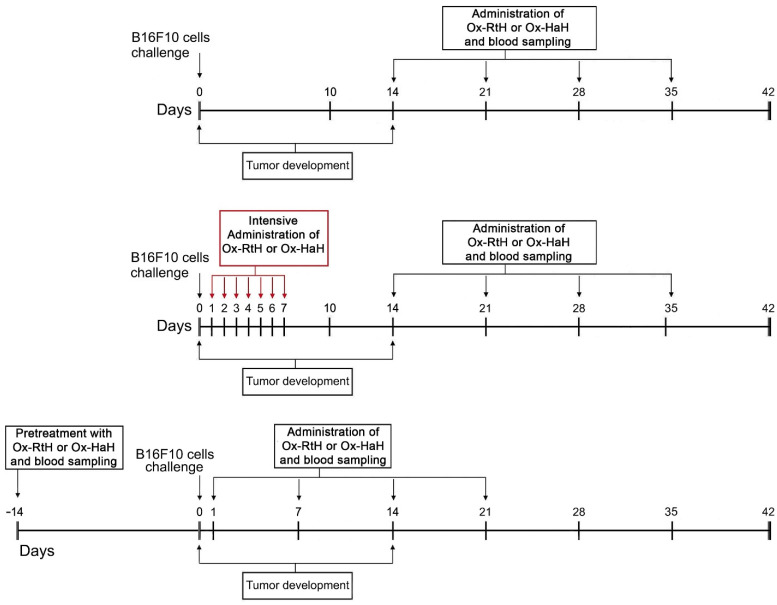
Experimental design and scheme for treatment of the test groups.

**Figure 2:**
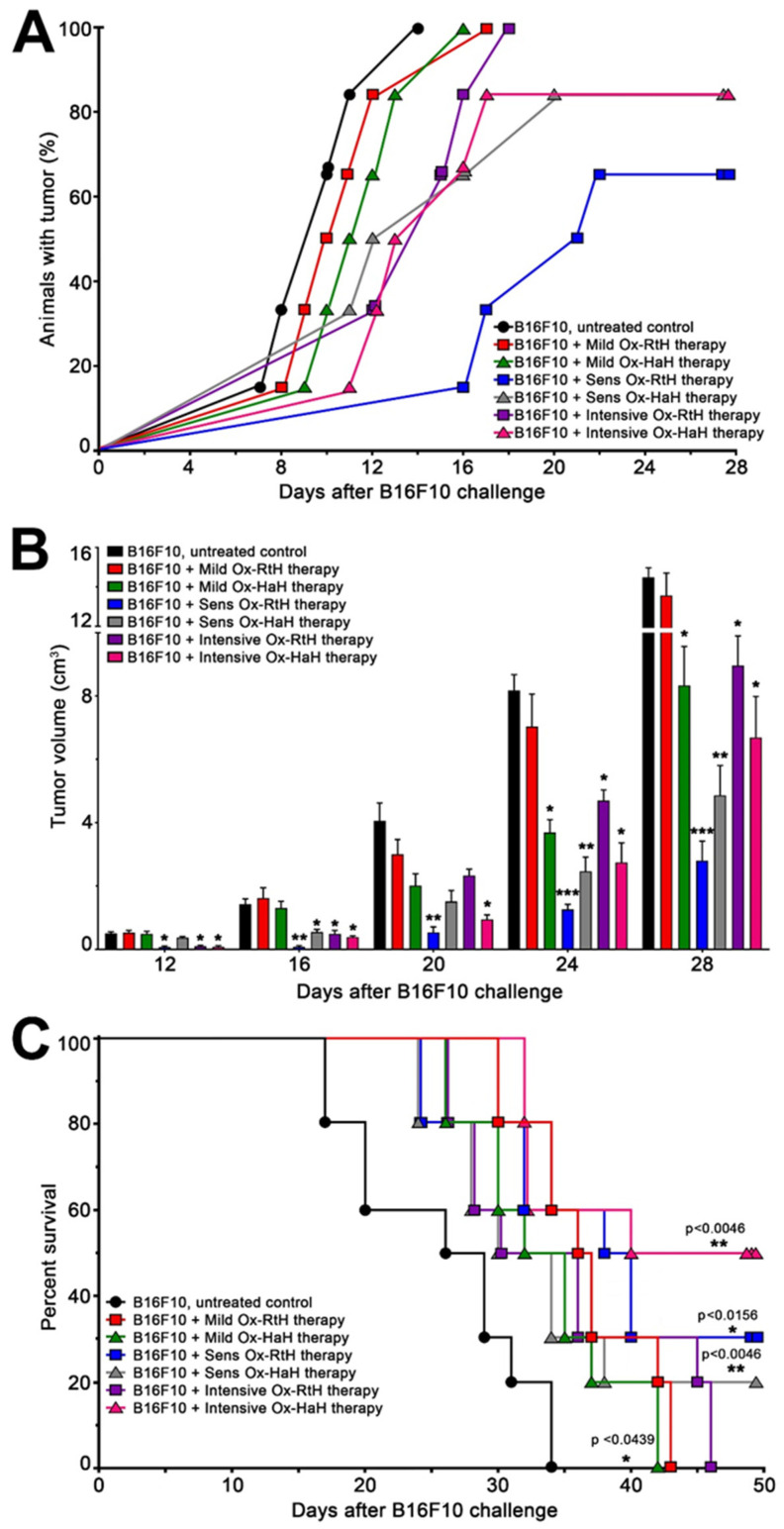
Tumor incidence, tumor development and survival of tumor-bearing mice under three regimens of treatment with Ox-RtH and Ox-HaH in the B16F10 murine melanoma model. (**A**) Tumor incidence in the Ox-Hcs-treated animals under the different immunization schemes. (**B**) The effect of Ox-Hcs therapy on tumor growth was monitored, and the sizes of solid tumors in all experimental groups (*n* = 10 mice each) were measured and compared with the tumor-bearing control group. Values in figures represent mean ± SD; *p* values were calculated using a two-way ANOVA test to determine the differences between any two groups (* *p* < 0.05, ** *p* < 0.01, *** *p* < 0.001). (**C**) Survival of Ox-Hc-treated animals was monitored and analyzed in all experimental groups (n = 10 mice each). Significance was determined through analysis of survival curves using the Kaplan and Meier method; *p*-values were calculated (* *p* < 0.05; ** *p* < 0.01) in comparison with B16F10-bearing mice. Representative data of 3 independent experiments are shown.

The challenge of animals with B16F10 tumor cells and the schedules for the Hcs treatment of the respective groups is shown in Figure 1. All animals in the untreated control group developed palpable solid tumors 14 days after challenge with B16F10 cells. Notably, mice subjected to immunization with both Ox-Hcs showed delayed tumor development, with 30% of animals in the intensive Ox-RtH and 20% in the intensive Ox-HaH and sensitized Ox-HaH groups exhibiting no tumor development by day 28 (Figure 2A).

The study findings indicate that both Ox-Hcs effectively inhibit tumor growth in the sensitized and intensive therapeutic approaches (Figure 2B). The groups that underwent both treatments showed a powerful suppression of tumor growth and development, while the mild treatments were less effective compared with them.

Survival curves showed similar trends for both Hcs (Figure 2C). All control mice died by day 34, whereas animals pretreated with either Ox-RtH or Ox-HaH had survival rates of 20% and 30% by day 50, respectively. The best survival rate was shown by the mice intensively treated with Ox-HaH, where 50% of the animals were kept alive during the observed period. No toxic effects were observed in mice during the studies with either Hcs.

### 2.3. Phenotypic Analysis of Tumor Infiltration

**Figure 3:**
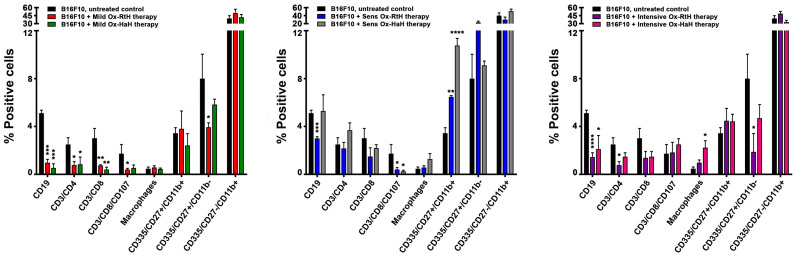
Phenotypic Analysis of Tumor Infiltration. The resulting data from all experiments are graphically presented as the percentage of viable immune cells. The data are expressed as mean ± SD for each group (n = 6–14), *p* values were calculated using the two-way ANOVA test (* *p* < 0.05; ** *p* < 0.01; *** *p* < 0.001; **** *p* < 0.0001) in comparison with B16F10-bearing mice.

We used FACS to perform quantitative analysis of the tumor microenvironment in C57BL/6 mice challenged with B16F10 cells. The investigation aimed to evaluate the impact of immunization with Ox-RtH and Ox-HaH using different administration approaches. We found significant differences in tumor-infiltrating lymphocytes after immunization with both Hcs, compared with untreated tumor-bearing controls (Figure S1, Figure 3).

Following a mild treatment regimen with Ox-RtH or Ox-HaH, we found a significant reduction in the percentage of tumor-infiltrating immune cells in all specific cell populations compared with the control group. For most of the populations, such as CD19+ B cells, CD4+, CD8+ and activated CD8+ T cells, the differences were statistically significant, while significance for NK cells at an early stage of maturation (CD27+CD11b-) was found only in the group treated with Ox–RtH (Figure 3, left).

Significant differences were observed in some cell populations in the sensitized groups treated with Ox-Hcs. A significant increase in the percentage of positive mature NK cells (CD27+CD11b+) was found in both groups of mice treated with Ox-RtH and Ox-HaH compared with untreated tumor-bearing animals, while a significant decrease was measured in activated CD8+ T cells in both groups (Figure 3, middle). Furthermore, significant decrease was also detected for B cells in the Ox-RtH-treated group.

After intensive treatment of the experimental animals with both Ox-Hcs, a significant reduction was measured in the B cell population, as well as for CD4+ T cells and immature NK CD27+CD11b- cells (significant only in the Ox-RtH group). A significant increase in the percentage was found only for the macrophages in the Ox-HaH-immunized animals, compared with the controls (Figure 3, right).

### 2.4. M1/M2 Macrophages in the Tumor Microenvironment

**Figure 4:**
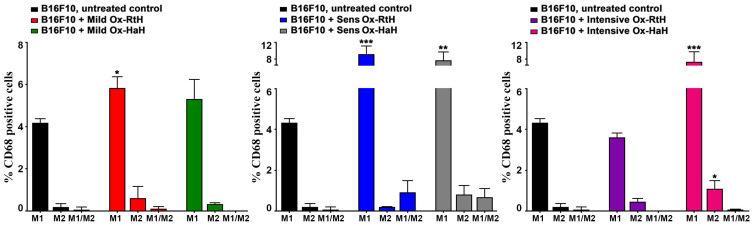
Extracted graphical results as the percentage of total CD68-positive tumor-infiltrating macrophages. FACS analyzed thirty thousand cells from each sample, which were monitored for M1/M2 discrimination. The data are presented as mean ± SD for each group (n = 6–14), *p* values were calculated using the two-way ANOVA test (* *p* < 0.05; ** *p* < 0.01; *** *p* < 0.001) in comparison with B16F10-bearing mice. Representative data from 3 independent experiments are shown.

To determine whether both Ox-Hcs promote M1/M2 polarization of macrophages in the tumor tissue, we used FACS to measure the distribution of F4/80+CD86+ (M1) and F4/80+CD206+ (M2) in a single-cell suspension, prepared from a tumor (Figure 4). In the mild therapy groups, significantly higher levels of M1 TAMs were observed in mice injected with both Ox-Hcs compared with untreated tumor-bearing animals, but the increase was significant only for animals treated with Ox-RtH. Furthermore, significantly higher values for M1 TAMs were also detected in the Sens-Ox-RtH or Sens-Ox-HaH groups compared with control animals. The intensive Ox-RtH therapy group had a non-significantly lower number of M1-like TAMs. On the contrary, Ox-HaH treatment led to significantly higher levels of M1 TAMs in the animal groups receiving the same intensive therapy compared with mice challenged with tumor cells only. Non-significant changes in M2 levels were observed in all groups injected with both Ox-Hcs, but significance was found only in animals intensively treated with Ox-HaH. Non-significantly increased values of double-positive M1/M2 macrophages were found in the groups of tumor-bearing mice pretreated with Ox-Hcs.

### 2.5. Evaluation of Tumor-Specific IgM Antibodies

**Figure 5:**
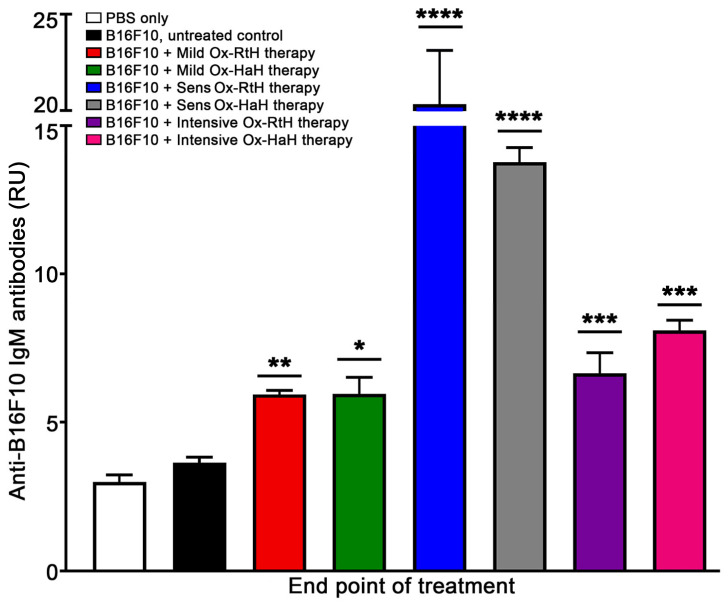
Detection of IgM antibodies against B16F10 generated in the experimental mice after treatment with Ox-RtH or Ox-HaH under the three regimens of administration. IgM levels in the sera were measured using ELISA, as detailed in the Section 4. Materials and Methods. Each sample was tested in triplicate and the results are displayed in relative units (RUs). The average values for each sample were used for the analysis and mean ± SD values were calculated for each group (n = 6–14) using Dunnett’s multiple comparisons test (* *p* < 0.05; ** *p* < 0.01; *** *p* < 0.001; **** *p* < 0.0001), compared with the control group of B16F10-bearing mice.

At the last stage of the experiments, blood samples were collected from all animals and sera were individually tested by ELISA to detect anti-B16F10 IgM antibodies. The mice from all Ox-Hcs-treated groups generated higher levels of anti-B16F10 IgM antibodies compared with the PBS-control group or B16F10-challenged untreated group. The highest significant values were detected among animals in the Sens-Ox-RtH and Sens-Ox-HaH groups compared with the control group (Figure 5).

### 2.6. CTL Activity

**Figure 6:**
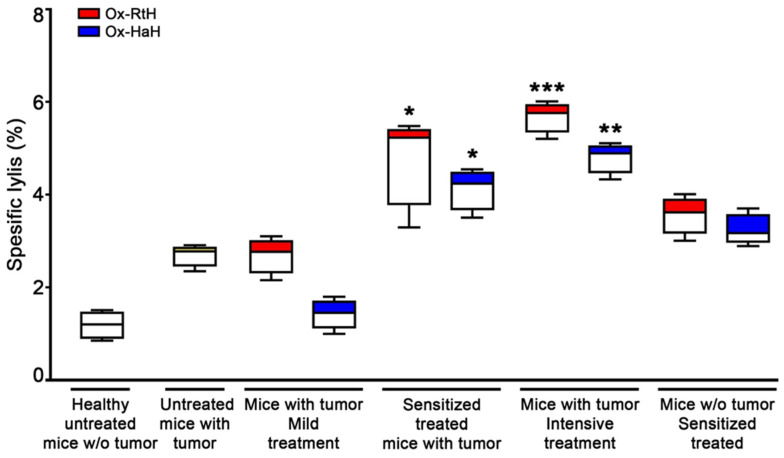
Tumor-specific cytotoxic response by CD8+ T-lymphocytes isolated from the spleen of experimental mice at the end poind of treatment with Ox-RtH or Ox-HaH. The generated CTL activity against B16F10 melanoma target cells was evaluated as a reference concentration of lactate dehydrogenase (LDH) released into the culture medium due to target cell lysis. All samples were triplicated and the average values were used for the analysis. The data are presented as mean ± SD for each group; p values were calculated using the two-way ANOVA test (* *p* < 0.05; ** *p* < 0.01; *** *p* < 0.001) compared to the control group of B16F10-bearing mice. Representative data of 3 independent experiments are shown.

To assess the ability of Ox-RtH and Ox-HaH to stimulate B16F10 cell-specific CTLs under different treatment regimens, we performed a specific CTL activity assay. Splenocytes were isolated from all experimental animals and used as effector cells against B16F10 melanoma tumor cells. Lactate dehydrogenase (LDH) release was measured spectrophotometrically after specific lysis by CTLs of target tumor cells. Groups intensively treated with both Ox-Hcs generated the highest statistically significant cytotoxic values, and animals treated with Ox-RtH performed better than the group immunized with Ox-HaH. A significantly higher percentage of CTLs was found in both Sens-Ox-Hcs groups compared with untreated tumor-bearing controls (Figure 6).

### 2.7. Cytokine Assay

**Figure 7:**
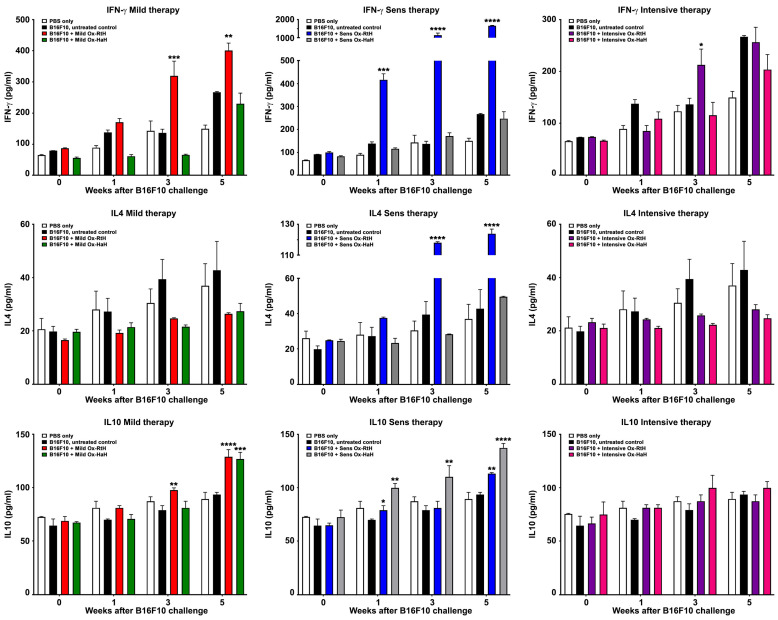
Analysis of cytokine production. Levels of IFNγ, IL4 and IL10 were quantified in mouse sera from experimental mice after treatment with Ox-RtH or Ox-HaH. Each sample was tested in triplicate, and the mean values were utilized for subsequent analysis. The data are presented as mean ± SD for each group (n = 6–14). Statistical significance was determined using a two-way ANOVA test, with *p* values as follows: * *p* < 0.05; ** *p* < 0.01; *** *p* < 0.001; **** *p* < 0.0001, compared with B16F10-bearing mice without treatment. The results display representative data from 3 independent experiments.

The levels of IFNγ, IL4, and IL10 in the weekly collected sera of C57BL/6 mice were measured in all experimental groups using quantitative ELISA (Figure 7). The administration of Ox-RtH in the mild-treated groups resulted in significantly higher production of IFNγ and IL10 at the 3rd and 5th week after challenge with B16F10 cells, when compared with the control tumor-bearing group. However, the same therapy did not result in significant differences between the values of the Ox-HaH-treated test groups and controls with only one significant result for IL 10 at week 5. No significant differences for IL4 production were found between the groups under the mild regiment of therapy.

The pretreatment of B16F10 cell-challenged mice with Ox-RtH significantly increased the serum levels of all measured cytokines compared with controls, whereas the sensitization of animals with Ox-HaH resulted in significantly higher levels of IL10 only.

No valuable changes were measured between the groups under the intensive scheme of treatment with Ox-Hcs. Significant increase was found only for one measurement point of IFNγ value after therapy with Ox-RtH.

## 3. Discussion

Melanoma is a highly aggressive form of skin cancer and is responsible for most skin cancer-related deaths. Melanoma incidence and mortality rates vary among ethnic groups. New biological therapies have gained prominence due to their targeted effectiveness and minimal side effects, replacing conventional treatments such as chemotherapy and radiotherapy. Immunotherapy, which works through the interaction between the immune system and cancer cells, includes the use of monoclonal and chimeric antibodies approved for clinical trials in the treatment of melanoma. This promising approach, including monoclonal antibodies for the therapy of melanoma, targets the programmed cell death protein 1 receptor (PD-1) and its ligand (PDL-1), cytotoxic T-lymphocyte-associated protein 4 (CTLA-4) and lymphocyte-activation gene 3 (LAG-3) in order to enable the tumor-silenced immune response [23]. However, current melanoma treatments are unable to effectively detect and destroy tumors, which has prompted ongoing research to discover new molecules and mechanisms for successful cancer therapy [1,24].

Natural products deliver new molecules that can potentially boost the immune system for tumor recognition. For instance, the giant oxygen-carrier glycoproteins called hemocyanins, which can be isolated from mollusks and gastropods, have shown beneficial immunological effects. These Hcs have been approved as potent protein carriers for antibody production and are a key component of anticancer therapeutic vaccines. Their complex structure is organized via decamers or multi-decamers with huge molecular mass. Hcs consist of 10 subunits (330 to 550 kDa each) that contain seven or eight globular functional units, each carrying two copper ions with the potential to reversibly bind one oxygen molecule. Due to their xenogeneic origin with evolutionary distance, immunization of mammals with Hcs elicits a very potent innate and adaptive immune response [19,25,26].

Hcs, characterized by their highly immunogenic nature that can be attributed to specific oligosaccharide compositions, including mannose and fucose, interact with C-type lectin receptors, namely the mannose receptor (MR) and toll-like receptor (TLR) 4, found on both dendritic cells (DCs) and macrophages, activating innate immunity. Recent studies have shown that KLH activates and matures human dendritic cells, and that this property is partially mediated by the mannose receptor engaged by mannose and fucose ligands carried by KLH. Similar effects have been observed with CCH and FLH, whose mannose receptor-mediated endocytosis has been found in murine peritoneal macrophages [8,27]. This activation leads to increased expression levels of certain molecules and secretion of specific interleukins and tumor necrosis factor [28,29,30,31]. Upon activation, antigen-presenting cells (APCs) induce the production of proinflammatory cytokines and signal internalization, promoting interaction with T and B lymphocytes and eliciting specifically targeted cellular and humoral immune responses [32,33]. The discovered properties of Hcs have facilitated antitumor studies in various mouse cancer models, forming the basis for the development of numerous therapeutic anticancer vaccines.

In recent years, a series of experiments elucidating the antitumor and immunostimulatory properties of sodium periodate-oxidized forms of Hcs have shown contradictory results. The oxidized form of CCH (Ox-CCH) exhibits more pronounced immunostimulatory properties as a protein carrier than the natural form of Hc. Furthermore, Ox-CCH showed a significant antitumor effect in the B16F10 melanoma model, similar to that induced by CCH, by inducing IFN-γ secretion. These findings suggest that periodic oxidation of the sugar moieties stabilizes the CCH structure, forming a more stable conformation and thereby enhancing its adjuvant and immunostimulatory effects. In contrast, several studies have indicated that treatment with Oxidized-FLH (Ox-FLH) resulted in tumor growth retardation in a mouse melanoma model, though the survival rate was significantly reduced after deglycosylation. However, Ox-FLH treatment led to a slightly higher antibody titer. Furthermore, a reduced capacity to activate dendritic cells was observed from the deglycosylated form in contrast with native FLH. This suggests that the anti-tumor effect of Ox-FLH is less durable and that the oligosaccharides and/or structural features affected by the chemical treatment of FLH are fundamental for its anti-tumor effects [8,9,20,34,35].

The behavior of each Hc appears to be a consequence of individual specificity in their compositions, despite their similar protein structure. Therefore, the contradictory results of analyzing different Hcs are not isolated cases [9].

In our present study, we investigated the antitumor properties of modified Hcs using different administration schedules. The results show stimulation of both cellular and humoral immunity, with increased levels of specific IgM antibodies, CD8 + T cytotoxic cells and NK cells. Animal groups treated with Ox-RtH and Ox-HaH showed various outcomes in observed parameters, such as prolonged animal survival, reduced solid tumor volume and development of high levels of anti-B16F10 IgM antibodies. These were found to be more pronounced in the group with pretreatment with both Ox-Hc, when compared with untreated control animals. Anti-B16F10 antibodies secreted by tumor-infiltrating B cells are expected to enhance the effects of CD8+ and NK cell infiltration through the mechanisms of antibody-dependent cellular cytotoxicity and by activating the complement system [36,37]. In our study, the generation of significantly high titers of anti-B16F10 IgM antibodies in the groups of immunized mice with both Ox-Hcs contributed to in vivo antitumor effects due to their recognition of surface antigens on B16F10 cells.

Macrophages, like other immune cells, have multiple subtypes that are activated under the influence of various stimuli from the microenvironment, including cytokines. Consequently, they modulate their transcriptional profile and function in response to tissue inflammatory challenge, polarizing into two different subtypes with pro- and anti-inflammatory properties, named M1-like (classically activated) and M2-like (alternatively activated) macrophages, respectively. The primary factor activating pro-inflammatory M1 macrophages is IFN-γ, a soluble cytokine produced by activated CD4+ T-helper (Th1) cells, CD8+ T cytotoxic cells, and NK cells. IFN-γ activates resting macrophages (M0) so that they become potent cells with increased antigen-presenting capacity, an enhanced ability to synthesize pro-inflammatory cytokines and toxic mediators, and allow for mediated phagocytosis. Conversely, M1 macrophages stimulate the Th1 response and cytotoxic cells, which, in turn, release IFN-γ, thereby enhancing M1 polarization. M1 macrophages express proinflammatory cytokines, including IL-1β, IL-6, IL-12, IL-23, IFN-β, and TNF-α, as well as proinflammatory chemokines (CXCL9, CXCL10, and CXCL15), which together drive Th1 responses and are also a major source of IL-12 [38,39].

We found high values of M1 macrophages in the various therapeutic approaches with Ox-Hc, correlating with high values of tumor-specific CTLs. Additionally, animals injected with Ox-RtH exhibited high serum levels of IFN-γ, fostering the cell immunity. Local intratumoral production of IFN-γ by tumor-infiltrating NK, CD4 Th1, and CD8 cytotoxic T lymphocytes appears to result in the generation of high levels of specific antitumor CTLs, ultimately leading to tumor growth suppression.

The role of IL-4 in tumor immunity presents a paradox. Initial mouse experiments demonstrated potent antitumor effects of IL-4, surpassing other cytokines in prophylactic and therapeutic models. Indeed, cancer patients often exhibit elevated levels of IL-4 in the tumor microenvironment. Both peripheral blood lymphocytes and tumor-infiltrating lymphocytes demonstrate a predominant polarization toward the secretion of IL-4 and other Th2 cytokines [40]. The presence of IL-4 enhances tumor metastasis and the highly metastatic variant of B16 melanoma (B16F10) induces a distinct CD4 T cell subset capable of strong IL-4 production compared with T cells induced by the low metastatic variant (B16F1). Systemic administration of IL-4 enhanced B16F1 metastasis to match that of B16F10. In contrast, neutralization of IL-4 by an IL-4-specific mAb reduced B16F10 metastases to the level of B16F1 [41].

Our experimental findings have shown a significant increase in IL-4 levels in the serum of tumor-bearing mice. These higher levels are associated with immune cells infiltrating the tumor and the continued presence of M2-type macrophages. We also found higher levels of M2 and IL-4 in the Ox-RtH pretreated group.

The results obtained from the treatment with two modified forms of Hcs under different regimens are promising, but various factors influence the different pathological indicators. The use of Ox-Hcs with distinct origin leads to varying results between different treatment regimens. Treatment of a murine melanoma model with Ox-CCH and Ox-FLH showed that the behavior of each Hc variant was due to the individual oligosaccharides in their content, despite their similar protein structure. The results of the studies confirm the positive immune response to the two modified Hcs and validate the results obtained on Ox-CCH [8].

In our study, both tested modified Hcs Ox-RtH and Ox-HaH induced a strong antitumor immune response after treatment of B16F10 tumor-bearing mice under different immunization regimens. Effective antitumor therapy is highly dependent on immunization regimens and Hc selection, indicating the need for future research in human clinical trials.

## 4. Materials and Methods

### 4.1. Antibodies

The following antibodies were used for fluorescence-activated cell sorting (FACS) experiments: anti-mouse Fluorescein isothiocyanate (FITC)-conjugated CD8 and CD335; Pacific Blue-conjugated CD19; Phycoerythrin (PE)-conjugated F4/80, CD107a, CD68, and CD27; Allophycocyanin (APC)-conjugated CD4, CD86, and CD11b; Brilliant Violet 421-conjugated CD163; and eFlour450-conjugated CD45 and CD3 mAbs (eBioscience, Frankfurt, Germany). Alkaline phosphatase (AP)-labeled anti-mouse IgM Ab (Sigma-Aldrich, Taufkirchen, Germany) was used for enzyme-linked immunosorbent assay (ELISA).

### 4.2. B16F10 Cell Line Culturing and Lysate Preparation

The murine melanoma cell line B16F10 (ATCC^®^ CRL6475 ™) was kindly provided by Dr. Sergej Tomic, Institute for the Application of Nuclear Energy (INEP), University of Belgrade. Cell culture, maintenance, single-cell suspension and lysate preparation of B16F10 cells were performed as described [16].

### 4.3. Animals

Female 6 weeks old C57BL/6 mice were purchased from The Jackson Laboratory (Bar Harbor, ME, USA). Animals were maintained under specific pathogen free (SPF) conditions at 22 °C with a 12 h light/dark cycle at the Institute of Microbiology, Bulgarian Academy of Sciences. The animal experiments and manipulations were conducted in strict adherence to the guidelines for the Care and Use of Laboratory Animals of the European Union (EU Directive 2010/63/EU) and approved by the Animal Care Commission at the Bulgarian Food Safety Agency (BFSA) (N286/16.04.2021) in compliance with national regulations.

### 4.4. Isolation, Purification and Chemical Modification of Hcs

The RtH and HaH were isolated and purified from the hemolymph of *Rapana thomasiana* and *Helix aspersa*, respectively, as previously described [16,42,43]. Further, the residual endotoxins were assessed in both Hcs using Limulus Amebocyte Lysate coatest gel (LAL) (Chromogenix AB, Molndal, Sweden).

The chemical oxidation method using sodium periodate was used to modify RtH and HaH according to the procedure described before [21,22]. Briefly, each Hc (2 mg/mL) was dissolved in 0.1 M sodium acetate buffer (pH 5.5), supplemented with 15 mM sodium periodate, and was subsequently incubated for 1 h in the dark at room temperature. Later, 25 μL of ethylene glycol was added for each 2 mL of protein solution and left to incubate overnight at 4 °C. Finally, the protein samples (Ox-RtH and Ox-HaH) were concentrated through ultrafiltration (Amicon 840 ultrafiltrator unit), followed by dialysis against 50 mM Tris-HCl buffer pH 8.0, and filtration through 0.22 μm membrane filter.

### 4.5. Tumor Induction in Mice and Treatment Schedule

Female 10 weeks old C57BL/6 mice were divided into ten groups (thirty animals each). Ten mice from each group were used for survival analysis, while the remaining animals were included in ex vivo tests. The tumors were induced in mice by subcutaneous (s.c.) challenge into the right flank with a single-cell suspension of murine melanoma cells B16F10 (1.5 × 10^5^ cells/mouse). After formation of palpable solid tumor, two animal groups were injected weekly with either 100 μg/mouse of Ox-RtH (mild Ox-RtH group) or Ox-HaH (mild Ox-HaH group) directly administered to the tumor site for 4 weeks (Figure 1).

Another two groups of mice were treated s.c. intensively with 100 μg/mouse of Ox-RtH or Ox-HaH in the area of B16F10 cell inoculation for 7 consecutive days, starting the day after the tumor cells challenge (intensive Ox-RtH and intensive Ox-HaH groups). This therapy was prolonged by weekly intratumoral injection of the same amounts of Ox-RtH or Ox-HaH for 4 weeks.

The other two groups were sensitized s.c. with Ox-RtH or Ox-HaH (100 μg/mouse) 14 days prior to the B16F10 cell challenge, labeled as the Sens-Ox-RtH and Sens-Ox-HaH groups. Subsequently, mice underwent weekly immunizations with the same doses of Ox-RtH or Ox-HaH at the site of tumor cell inoculation for 4 weeks, starting the day after challenge with the tumor cells.

Two additional control groups of mice were subjected to immunization with either Ox-RtH or Ox-HaH, without challenge by B16F10 cells under the sensitized regimen of treatment. Another control group received injections of PBS only, also without exposure to tumor cells. The last control group of mice was exposed to B16F10 cells and subsequently injected with PBS. The animals from all groups were bled weekly from the retro-orbital sinus, and the collected sera were stored frozen at -70 °C for further analyses.

### 4.6. Tumor Assessment and Organ Collection

The incidence and progression of tumor development, as well as survival outcomes, were assessed in accordance with previously established protocols [16,18]. Tumor volumes were calculated using the following formula:

Volume (cm^3^) = width^2^ × length × 0.52

The animal groups were monitored over a 7-week period, and the survival rates of the treated tumor-bearing groups were compared with those of the untreated B16F10 cells challenged group. A second quantity of experimental animals from each group was sacrificed on day 29 after being challenged with B16F10 cells, and the solid tumors and spleens were collected from all mice. Only for the control tumor-bearing group were the mice doubled because of the low survival rate in this point.

### 4.7. Flow Cytometry Analyses of Tumor Infiltration

The analysis of tumor-infiltrated lymphocytes and immune cell phenotype determination was performed using FACS. Solid tumors from all terminal animals were removed and single-cell suspensions were prepared by grinding through sterile cell strainers. Tumor samples were distributed in FACS tubes (2 × 10^5^ cells/tube) and incubated for 20 min on ice with one of the following mixes of anti-mouse antibodies in order to identify different cell populations: B (CD19-PE), T (CD3e-eFlour450/CD4-APC and CD3e-eFlour450/CD8-FITC/CD107a-PE), activated T (CD3+CD8+CD107+), macrophages (CD11b-APC/F4/80-PE), and NK cells (CD3e-eFlour450/CD335-FITC/CD11b-APC/CD27-PE). Thirty thousand cells were analyzed from each sample with a BD LSR II flow cytometer (BD Biosciences, San Jose, CA) using Diva 6.1.1. software (BD Biosciences, Mountain View, CA, USA).

### 4.8. M1/M2 Macrophage Phenotyping

Analysis of tumor-associated macrophages (TAMs) and assessment of the ratio between M1 and M2 subtypes was performed using FACS as already described [16]. Single-cell suspensions from tumors (as described above) were subjected to washing and distribution into FACS tubes, followed by incubation with either anti-mouse CD68-PE/CD86-APC or CD68-PE/CD163-Brilliant Violet 421 antibodies. Based on gated CD68-positive cells, the M1 and M2 phenotype of macrophages was determined with a BD LSR II flow cytometer using the Diva 6.1.1. software.

### 4.9. ELISA for Detection of IgM Antibodies against B16F10 Cells

The levels of IgM antibodies specific for B16F10 cells were evaluated by ELISA as previously described [18].

### 4.10. Cytotoxic T-lymphocyte Assay

The presence of induced tumor-specific cytotoxic T lymphocytes (CTLs) was assessed using a non-radioactive cytotoxicity assay kit (CytoTox, Promega, Madison, WI, USA) as previously described [16]. Briefly, cultured B16F10 cells were detached from an 80% confluent cell monolayer using accutase (eBioscience) and cell strainers (BD Biosciences, Erembodegem, Belgium). Then, a single-cell suspension was transferred to a 96-well culture plate (1 × 10^4^ cells/well). The cells were cultured in complete Dulbecco’s Modified Eagle Medium (DMEM, Gibco, Gaithersburg, MD, USA) supplemented with 10% heat-inactivated fetal calf serum (FCS), 1 mM sodium pyruvate, 4 mM L-glutamine, and antibiotics at 37 °C/5% CO_2_.

Spleens from sacrificed animals were removed from all experimental mice and the splenocytes were isolated by sterile cell strainers for single-cell suspension preparation. After lysis of erythrocytes with hypotonic buffer, cells were counted with a hemocytometer and used as effector cells in a cytotoxic assay. The splenocytes were added to the wells (4 × 10^5^ cells/well) at a ratio of 1:40 with the target B16F10 cells and the specific lysis (%) was determined according to the commercial kit instructions.

### 4.11. Cytokine Detection

The serum levels of interferon gamma (IFNγ), interleukin 4 (IL4), and interleukin 10 (IL10) were assessed using ELISA MAXTM Deluxe Set (BioLegend, Waltham, MA, USA) according to the manufacturer’s instructions.

### 4.12. Statistical Analysis

All statistical analyses were performed with Prism 10 software from GraphPad (San Diego, CA, USA). The two-way ANOVA test was used to determine differences between each two groups. Values in the figures correspond to mean ± SD and a value of *p* < 0.05 was considered as statistically significant. ELISA, cytokine, and cytotoxicity samples were tested in triplicate. The method of Kaplan and Meier was used to determine survival significance.

## Data Availability

The data presented in this study are available on request from the corresponding author.

## Supplementary Materials

The following supporting information can be downloaded at: https://www.mdpi.com/article/10.3390/md22100462/s1, Figure S1: Flow cytometry for phenotyping of tumor-infiltrating immune cells.

## References

[1] Centeno P.P., Pavet V., Marais R. (2023). The journey from melanocytes to melanoma. Nat. Rev. Cancer.

[2] Matthews N.H., Li W.Q., Qureshi A.A., Weinstock M.A., Cho E. (2017). Epidemiology of melanoma. Exon Publ..

[3] Zitvogel L., Pitt J.M., Daillère R., Smyth M.J., Kroemer G. (2016). Mouse models in oncoimmunology. Nat. Rev. Cancer.

[4] Isacescu E., Chiroi P., Zanoaga O., Nutu A., Budisan L., Pirlog R., Atanasov A.G., Berindan-Neagoe I. (2023). Melanoma Cellular Signaling Transduction Pathways Targeted by Polyphenols Action Mechanisms. Antioxidants.

[5] Juszczak A.M., Wöelfle U., Končić M.Z., Tomczyk M. (2022). Skin cancer, including related pathways and therapy and the role of luteolin derivatives as potential therapeutics. Med. Res. Rev..

[6] Naeem A., Hu P., Yang M., Zhang J., Liu Y., Zhu W., Zheng Q. (2022). Natural Products as Anticancer Agents: Current Status and Future Perspectives. Molecules.

[7] Mora Román J.J., Del Campo M., Villar J., Paolini F., Curzio G., Venuti A., Jara L., Ferreira J., Murgas P., Lladser A. (2019). Immunotherapeutic potential of mollusk hemocyanins in combination with human vaccine adjuvants in murine models of oral cancer. J. Immunol. Res..

[8] Arancibia S., Campo M.D., Nova E., Salazar F., Becker M.I. (2012). Enhanced structural stability of Concholepas hemocyanin increases its immunogenicity and maintains its non-specific immunostimulatory effects. Eur. J. Immunol..

[9] Arancibia S., Espinoza C., Salazar F., Del Campo M., Tampe R., Zhong T.Y., De Ioannes P., Moltedo B., Ferreira J., Lavelle E.C. (2014). A novel immunomodulatory hemocyanin from the limpet *Fissurella latimarginata* promotes potent anti-tumor activity in melanoma. PLoS ONE.

[10] Markl J., Lieb B., Gebauer W., Altenhein B., Meissner U., Harris J.R. (2001). Marine tumor vaccine carriers: Structure of the molluscan hemocyanins KLH and HtH. J. Cancer Res. Clin. Oncol..

[11] Moltedo B., Faunes F., Haussmann D., De Ioannes P., De Ioannes A.E., Puente J., Becker M.I. (2006). Immunotherapeutic effect of Concholepas hemocyanin in the murine bladder cancer model: Evidence for conserved antitumor properties among hemocyanins. J. Urol..

[12] Gesheva V., Idakieva K., Kerekov N., Nikolova K., Mihaylova N., Doumanova L., Tchorbanov A. (2011). Marine gastropod hemocyanins as adjuvants of non-conjugated bacterial and viral proteins. Fish Shellfish Immunol..

[13] Gesheva V., Chausheva S., Stefanova N., Mihaylova N., Doumanova L., Idakieva K., Tchorbanov A. (2015). Helix pomatia hemocyanin—A novel bio-adjuvant for viral and bacterial antigens. Int. Immunopharmacol..

[14] Gesheva V., Chausheva S., Mihaylova N., Manoylov I., Doumanova L., Idakieva K., Tchorbanov A. (2014). Anti-cancer properties of gastropodan hemocyanins in murine model of colon carcinoma. BMC Immunol..

[15] Stoyanova E., Mihaylova N., Manoylov I., Bradyanova S., Raynova Y., Idakieva K., Tchorbanov A. (2020). Intensive therapy with gastropodan hemocyanins increases their antitumor properties in murine model of colon carcinoma. Int. Immunopharmacol..

[16] Stoyanova E., Mihaylova N., Ralchev N., Ganova P., Bradyanova S., Manoylov I., Raynova Y., Idakieva K., Tchorbanov A. (2022). Antitumor Properties of Epitope-Specific Engineered Vaccine in Murine Model of Melanoma. Mar Drugs..

[17] Palacios M., Tampe R., Del Campo M., Zhong T.Y., López M.N., Salazar-Onfray F., Becker M.I. (2018). Antitumor activity and carrier properties of novel hemocyanins coupled to a mimotope of GD2 ganglioside. Eur. J. Med. Chem..

[18] Stoyanova E., Mihaylova N., Ralchev N., Bradyanova S., Manoylov I., Raynova Y., Idakieva K., Tchorbanov A. (2024). Immunotherapeutic Potential of Mollusk Hemocyanins in Murine Model of Melanoma. Mar. Drugs.

[19] Kato S., Matsui T., Gatsogiannis C., Tanaka Y. (2018). Molluscan hemocyanin: Structure, evolution, and physiology. Biophys. Rev..

[20] Salazar M.L., Jiménez J.M., Villar J., Rivera M., Báez M., Manubens A., Becker M.I. (2019). N-Glycosylation of mollusk hemocyanins contributes to their structural stability and immunomodulatory properties in mammals. J. Biol. Chem..

[21] Raynova Y., Todinova S., Idakieva K. (2017). Modification with sodium periodate increases the structural stability of molluscan hemocyanins. Bulg. Chem. Commun..

[22] Raynova Y., Yancheva D., Guncheva M., Idakieva K., Todinova S. (2019). Enhanced structural stability of oxidized Helix aspersa maxima hemocyanin. Curr. Top. Pept. Protein Res..

[23] Knight A., Karapetyan L., Kirkwood J.M. (2023). Immunotherapy in Melanoma: Recent Advances and Future Directions. Cancers.

[24] Kızılbey K., Türkoğlu N., Kırmızıtaş F.C. (2020). Immune System Modulations in Cancer Treatment: Nanoparticles in Immunotherapy. Cell Interaction-Molecular and Immunological Basis for Disease Management.

[25] Becker M.I., Arancibia S., Salazar F., Del Campo M., De Ioannes A. (2014). Mollusk hemocyanins as natural immunostimulants in biomedical applications. Immune Response Activation.

[26] Coates C.J., Nairn J. (2014). Diverse immune functions of hemocyanins. Dev. Comp. Immunol..

[27] Presicce P., Taddeo A., Conti A., Villa M.L., Della Bella S. (2008). Keyhole limpet hemocyanin induces the activation and maturation of human dendritic cells through the involvement of a mannose receptor. Mol. Immunol..

[28] Del Campo M., Arancibia S., Nova E., Salazar F., Gonzalez A., Moltedo B., De Ioannes P., Ferreira J., Manubens A., Becker M.I. (2011). Hemocyanins as immunostimulants. Rev. Médica Chile.

[29] Atanasov A.G., Zotchev S.B., Dirsch V.M., Supuran C.T. (2021). Natural products in drug discovery: Advances and opportunities. Nat. Rev. Drug Discov..

[30] Saeed A.F., Su J., Ouyang S. (2021). Marine-derived drugs: Recent advances in cancer therapy and immune signaling. Biomed. Pharmacother..

[31] Gianazza E., Eberini I., Palazzolo L., Miller I. (2021). Hemolymph proteins: An overview across marine arthropods and molluscs. J. Proteom..

[32] Zhong T.Y., Arancibia S., Born R., Tampe R., Villar J., Del Campo M., Manubens A., Becker M. (2016). Hemocyanins Stimulate Innate Immunity by Inducing Different Temporal Patterns of Proinflammatory Cytokine Expression in Macrophages. J. Immunol..

[33] Jiménez J., Salazar M., Arancibia S., Villar J., Salazar F., Brown G., Lavelle E., Martínez-Pomares L., Ortiz-Quintero J., Lavandero S. (2019). TLR4, but Neither Dectin-1 nor Dectin-2, Participates in the Mollusk Hemocyanin-Induced Proinflammatory Effects in Antigen-Presenting Cells from Mammals. Front Immunol..

[34] Pizarro-Bauerle J., Maldonado I., Sosoniuk-Roche E., Vallejos G., López M.N., Salazar-Onfray F., Becker M.I. (2017). Molluskan hemocyanins activate the classical pathway of the human complement system through natural antibodies. Front. Immunol..

[35] Ohmi Y., Kambe M., Ohkawa Y., Hamamura K., Tajima O., Takeuchi R., Furukawa K. (2018). Differential roles of gangliosides in malignant properties of melanomas. PLoS ONE.

[36] Díaz-Zaragoza M., Hernández-Ávila R., Viedma-Rodríguez R., Arenas-Aranda D., Ostoa-Saloma P. (2015). Natural and adaptive IgM antibodies in the recognition of tumor-associated antigens of breast cancer (Review). Oncol. Rep..

[37] Dobroff A.S., Rodrigues E.G., Juliano M.A., Friaça D.M., Nakayasu E.S., Almeida I.C., Mortara R.A., Jacysyn J.F., Amarante-Mendes G.P., Magliani W. (2010). Differential Antitumor Effects of IgG and IgM Monoclonal Antibodies and Their Synthetic Complementarity-Determining Regions Directed to New Targets of B16F10-Nex2 Melanoma Cells. Transl. Oncol..

[38] Masuda J., Shigehiro T., Matsumoto T., Satoh A., Mizutani A., Umemura C., Saito S., Kijihira M., Takayama E., Seno A. (2018). Cytokine expression and macrophage localization in xenograft and allograft tumor models stimulated with lipopolysaccharide. Int. J. Mol. Sci..

[39] Gonzalez H., Hagerling C., Werb Z. (2018). Roles of the immune system in cancer: From tumor initiation to metastatic progression. Genes Dev..

[40] Li Z., Chen L., Qin Z. (2009). Paradoxical roles of IL-4 in tumor immunity. Cell. Mol. Immunol..

[41] Kobayashi M., Kobayashi H., Pollard R.B., Suzuki F. (1998). A pathogenic role of Th2 cells and their cytokine products on the pulmonary metastasis of murine B16 melanoma. J. Immunol..

[42] Idakieva K., Chakarska I., Ivanova P., Tchorbanov A., Dobrovolov I., Doumanova L. (2009). Purification of hemocyanin from marine gastropod Rapana thomasiana using ammonium sulfate precipitation method. Biotechnol. Biotechnol. Equip..

[43] Raynova Y., Doumanova L., Idakieva K.N. (2013). Phenoloxidase activity of Helix aspersa maxima (garden snail, Gastropod) hemocyanin. Protein J..

