# Supplementary material for: Modified Hemocyanins from Rapana thomasiana and Helix aspersa Exhibit Strong Antitumor Activity in the B16F10 Mouse Melanoma Model
Source: Mar Drugs. 2024 Oct 7;22(10):462. doi: 10.3390/md22100462 (PMC11509262; doi:10.3390/md22100462)
Supplement: Supplementary file 1 [file marinedrugs-22-00462-s001.zip › marinedrugs-3222182-supplementary.pdf]

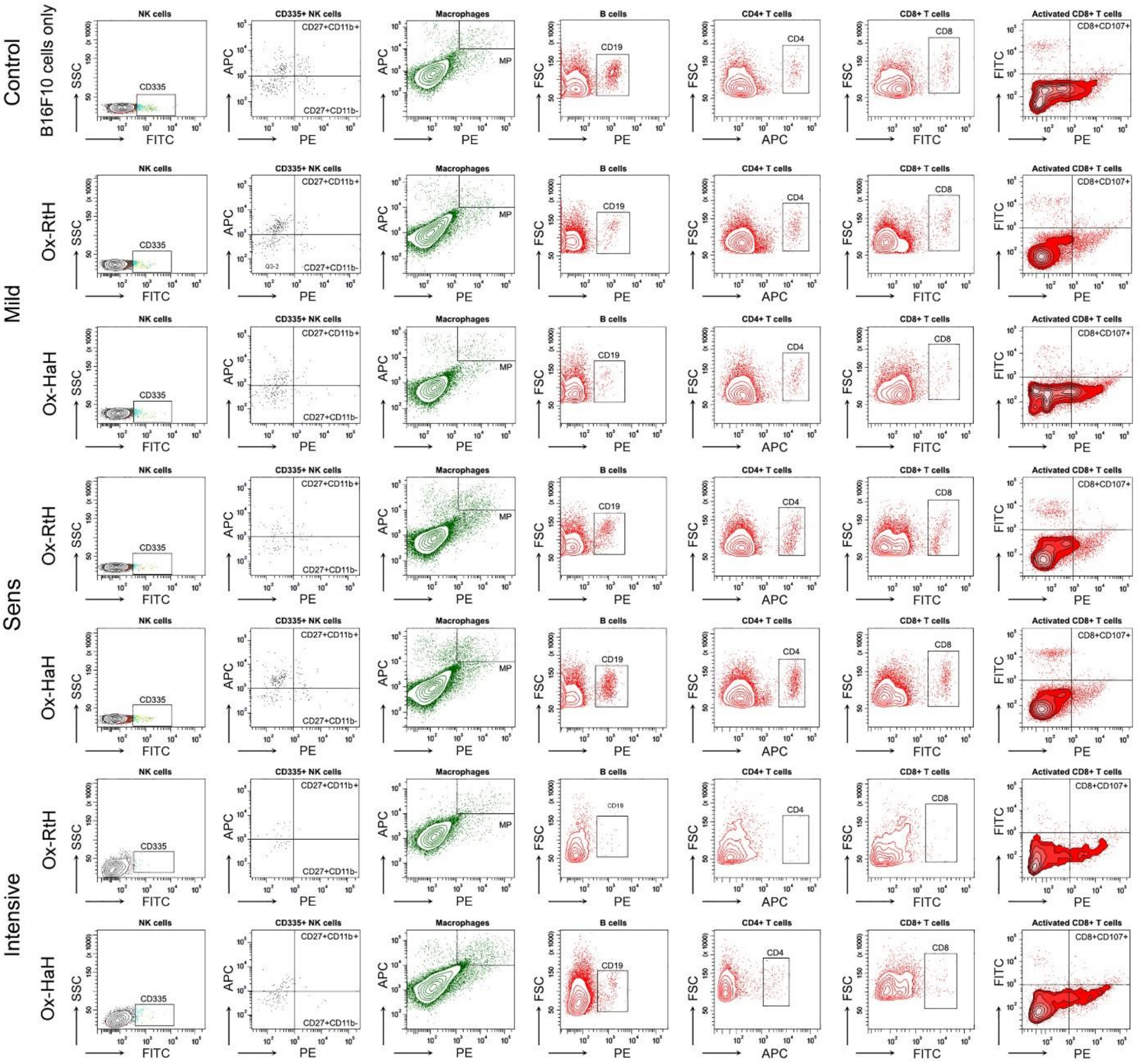

Figure S1. Flow cytometry for phenotyping of tumor-infiltrating immune cells was performed after treatment of mice with Ox-RtH and Ox-HaH. The quantitative analysis of tumor infiltration in solid tumors from all experimental animals was carried out via terminal animals (day 29 after B16F10 cells challenge). Tumor-derived single-cell suspensions were incubated with an appropriate mixture of fluorescent dye-labeled antibodies as described in the Section 4. Materials and Methods. Lymphocyte-gated cells from each sample (thirty thousand per tube) were then collected and analyzed using FACS. Representative images from three independent experiments.
